# Responsiveness and construct validity of EPIC-26, AQoL-6D and SF-6D following treatment in prostate cancer

**DOI:** 10.1186/s12885-023-10732-6

**Published:** 2023-04-01

**Authors:** Norma B. Bulamu, Christine Mpundu-Kaambwa, Michael O’Callaghan, Billingsley Kaambwa

**Affiliations:** 1 Flinders Health and Medical Research Institute, College of Medicine and Public Health, Flinders University, Adelaide, Australia; 2 Health and Social Care Economics Group, Caring Futures Institute, College of Nursing and Health Sciences, Flinders University, Adelaide, Australia; 3 SA-PCCOC: South Australian Prostate Cancer Clinical Outcomes Collaborative, Flinders Medical Centre, Urology Unit, Adelaide, Australia; 4 Discipline of Medicine, University of Adelaide, Adelaide, Australia

**Keywords:** Construct validity, Convergent validity, Discriminant validity, Responsiveness, EPIC-26, AQoL-6D, SF-6D, Prostate cancer

## Abstract

**Purpose:**

To assess construct validity and responsiveness of the Expanded Prostate Cancer Index Composite Instrument (EPIC-26) relative to the Short-Form Six-Dimension (SF-6D) and Assessment of Quality of Life 6-Dimension (AQoL-6D) in patients following treatment for prostate cancer.

**Methods:**

Retrospective prostate cancer registry data were used. The SF-6D, AQoL-6D, and EPIC-26 were collected at baseline and one year post treatment. Analyses were based on Spearman's correlation coefficient, Bland–Altman plots and intra-class correlation coefficient, Kruskal Wallis, and Effect Size and the Standardised Response Mean for responsiveness.

**Results:**

The study sample was comprised of 1915 patients. Complete case analysis of 3,697 observations showed moderate evidence of convergent validity between EPIC-26 vitality/hormonal domain and AQoL-6D (*r* = 0.45 and 0.54) and SF-6D (*r *= 0.52 and 0.56) at both timepoints. Vitality/hormonal domain also showed moderate convergent validity with coping domain of AQoL-6D (*r* = 0.45 and 0.54) and with role (*r* = 0.41 and 0.49) and social function (*r* = 0.47 and 0.50) domains of SF-6D at both timepoints, and with independent living (*r* = 0.40) and mental health (*r* = 0.43) of AQoL-6D at one year. EPIC-26 sexual domain had moderate convergent validity with relationship domain (*r* = 0.42 and 0.41) of AQoL-6D at both timepoints. Both AQoL-6D and SF-6D did not discriminate between age groups and tumour stage at both timepoints but AQoL-6D discriminated between outcomes for different treatments at one year. All EPIC-26 domains discriminated between age groups and treatment at both timepoints. The EPIC-26 was more responsive than AQoL-6D and SF-6D between baseline and one year following treatment.

**Conclusions:**

AQoL-6D can be used in combination with EPIC-26 in place of SF-12. Although EPIC-26 is not utility based, its popularity amongst clinicians and ability to discriminate between disease-specific characteristics and post-treatment outcomes in clinical trials makes it a candidate for use within cost-effectiveness analyses. The generic measure provides a holistic assessment of quality of life and is suitable for generating quality adjusted life years (QALYs).

**Supplementary Information:**

The online version contains supplementary material available at 10.1186/s12885-023-10732-6.

## Background

Globally, prostate cancer is the second most common cancer in men and one of the top four cancers overall [1, 2]. Australia is ranked among countries with the highest rates of prostate cancer, affecting up to 1 in 6 Australian men by age 85 and accounting for 25% of new cancer cases and 12% of cancer deaths among men [2, 3]. The associated healthcare cost of managing prostate cancer in Australia is estimated to rise by 42%, from US$270.9 million in 2016 to US$384.3 million in 2025 [4]. With rising costs, it is vital to demonstrate the benefits of the different treatments in terms of clinical effectiveness and health-related quality of life (HRQoL) through health economic modelling. Health economic evaluation is widely applied in the appraisal of health care interventions by comparing their costs and outcomes. Leading international organisations for health technology assessment such as the Pharmaceutical Benefits Advisory Committee (PBAC) in Australia and the National Institute for Health and Care Excellence (NICE) in the UK, recommend the use of cost-effectiveness analysis (CEA) and cost-utility analysis (CUA) as preferred methods of evaluation [5, 6]. Outcomes in CEA are measured in terms of clinical effectiveness and quality adjusted life years (QALYs) in CUA. A QALY is a measure of disease burden for use in health economic modelling that combines both the quantity of life attributed to a given health state or intervention and the value individuals place on that life when comparing two or more interventions [7]. QALYs are derived from responses to preference-based measures (PBM) of HRQoL. These measures comprise a descriptive system for classifying all possible health states defined by the instrument and an off-the-shelf scoring algorithm or value set comprising values on the 0 (dead) to 1 (full health) scale. Unlike clinical effectiveness metrics that are specific to a condition, the QALY is a generic measure that facilitates the comparison of outcomes between different disease conditions. Preference based measures can be generic such as the Short Form 6-Dimesnions, SF-6D [8] and Assessment of Quality of Life 6 dimension, AQoL-6D [9] or disease-specific, such as the cancer-specific PBMs derived from responses to the European Organization for Research and Treatment of Cancer core quality of life questionnaire (EORTC QLQ-C30), EORTC Quality of Life Utility Measure-Core 10 dimensions, QLU-C10D [10] and EORTC-8D [11] Other PBMs for specific conditions include the prostate cancer-specific Patient-Oriented Prostate Utility Scale (PORPUS) [12] and the patient-reported Short Bowel Syndrome-specific quality of life scale (SBS-QoL) [13].

Prostate cancer diagnosis and treatment affect both disease-specific and generic HRQoL, varying based on the stage of the disease, type of treatment received and time post-treatment. Men with localised disease experience disease-specific detriments to HRQoL, such as problems with urinary function, sexual function and bowel function but fewer problems with the generic HRQoL. Generic HRQoL is more affected in men with advanced disease, specifically physical functioning, social well-being, pain, and psychological well-being associated with the impending possibility of death at this stage [14–16]. Therefore, the challenge for quality of life measurement is that although generic HRQoL measures are suitable for generating utilities for application in CUA, they may not be as sensitive to changes in disease-specific quality of life [17–19]. Although disease-specific PBMs have been developed, when used in an economic evaluation, such results cannot be generalised or compared to other conditions, and therefore the need to still use the generic measures [20, 21]. However, the level of divergence in validity and responsiveness between disease-specific and generic PBMs when used in prostate cancer research is unclear.

Previous studies have assessed the construct validity of AQoL-6D and SF-6D in different patient populations [22, 23], but no studies have yet compared them to the Expanded Prostate Cancer Index Composite Instrument (EPIC-26), one of the most widely validated prostate cancer-specific measures of QoL [24]. This present study aimed to explore the construct (convergent and known groups validity and responsiveness of the generic preference-based HRQoL measures, SF-6D and AQoL-6D when compared to EPIC-26 in men with prostate cancer.

## Methods

### Data

The South Australian Prostate Cancer Clinical Outcomes Collaborative (SA-PCCOC) is a longitudinal dataset with linked public and private hospital admissions for approximately 17,000 men with prostate cancer since 1998. The database commenced in 1998 and currently recruits over 90% of newly diagnosed prostate cancer cases in South Australia, which has approximately 1.67 million people. The database captures information about patient demographics (age at diagnosis, date of diagnosis, postcode), diagnosis (cancer tumour stage), biochemistry (Gleason score and grade), treatment received (radical or palliative external beam radiation therapy—EBRT, brachytherapy, robot-assisted laparoscopic radical prostatectomy, open retropubic radical prostatectomy and active surveillance or watchful waiting) at baseline. HRQoL is assessed through self-completion of a paper-based survey that is mailed-out at the start of treatment or baseline, one year and 5-years post-treatment initiation. Data from the returned surveys is manually entered into the database. Patients over 18 years of age diagnosed with prostate cancer and commencing treatment between February 2010 and March 2020 were included in this study. HRQoL data on the Short Form Health Survey (SF-12) (from which the SF-6D was derived) [8], AQoL-6D [9] and the EPIC-26, collected at baseline and one year following treatment, were included in this study. Baseline measurements are taken between the date of diagnosis and the date of first treatment (or documented management plan). At any given time point, patients completed either EPIC-26 and SF-12 or EPIC-26 and AQoL-6D. No patients completed both generic measures simultaneously.

#### Measures/Instruments

The EPIC-26 is a prostate cancer-specific measure of HRQoL with five multi-item domains, namely urinary incontinence (four items), urinary irritation/obstruction (four items), bowel (six items), sexual (six items) and vitality/hormonal function (five items) and a single-item measure of overall urinary bother [25]. No total score for the entire measure can be calculated, but domain summary scores are generated on a 0–100 scale.

The AQoL-6D is a preference-based measure of quality of life with scoring algorithms or value sets based on Australian general adult and adolescent populations [9]. The AQoL-6D has six dimensions (independent living (four items), mental health, coping (four items), relationships (three items), pain (three items) and senses). Each dimension has five levels ranging from no difficultiescompleting tasks to severe difficulty. AQoL-6D domain and utility scores were generated based on the Australian adult general population scoring algorithm.

The SF-12 is a shorter version of the widely used SF-36 that includes only 12 items whose scores can be aggregated into two summary scales, the Physical Component Summary (PCS) and the Mental Component Summary (MCS) [8]. Although not preference-based, responses to the SF-12 can be used to generate SF-6D utility index scores using the algorithm developed by Brazier and Roberts [26]. Therefore, utility and six domain scores (for physical function, role function, social function, pain, mental health, and vitality) were generated from the SF-12.

### Analysis

AQoL-6D and SF-6D utilities and EPIC-26 domain summary scores were tested for normality using the Shapiro-Francia test [27] with parametric or non-parametric tests applied as appropriate.

#### Responsiveness

Responsiveness assesses whether an instrument detects change where change is expected over time. Responsiveness was assessed for patients who completed both baseline and one-year assessments for each instrument.

Two statistical tests were applied: Effect Size (ES) and the Standardised Response Mean (SRM) [28]. These tests measure the change in index scores relative to the variation among the sample or the average change in scores normalised by a measure of deviation, in this case, the standard deviation. Effect size demonstrates the extent to which an instrument measures changes in quality of life relevant to an intervention.

$$\begin{array}{c}\mathrm{Effect}\;\mathrm{size}=\frac{\mathrm{mean}\;\mathrm{change}\;\mathrm{from}\;\mathrm{baseline}\;\mathrm{to}\;\mathrm{one}\;\mathrm{year}}{\mathrm{standard}\;\mathrm{deviation}\;\mathrm{of}\;\mathrm{scores}\;\mathrm{at}\;\mathrm{baseline}}\\\mathrm{Standardised}\;\mathrm{Response}\;\mathrm{Mean}=\frac{\mathrm{mean}\;\mathrm{change}\;\mathrm{from}\;\mathrm{baseline}\;\mathrm{to}\;\mathrm{one}\;\mathrm{year}}{\mathrm{standard}\;\mathrm{deviation}\;\mathrm{of}\;\mathrm{the}\;\mathrm{mean}\;\mathrm{change}}\end{array}$$

For both ES and SRM, scores of < 0.20 represent a trivial effect, 0.20–0.49 a small effect, 0.50–0.80 a moderate effect and > 0.80 a large effect [29].

For these measures, we also assessed if the minimum clinically important difference (MCID) or clinically significant mean change was achieved over time. The MCID varies between EPIC-26 domains. A mean change of 4–6 points is considered clinically significant for the bowel and vitality/hormonal domains, a change of 10–12 points for the sexual domain, 6–9 points for urinary incontinence and 5–7 points for the urinary irritation/obstruction domain [30]. When used in general population samples, a mean change between baseline and follow-up of 0.13 or more reflect important differences between groups, and a change of 0.06 utility points over time is clinically significant for AQoL-6D [31], while a mean change of 0.012 for SF-6D has been reported in populations with incontinence, which may be similar to the sample in this study [32].

#### Construct validity

Construct validity “…refers to whether the instrument provides the expected scores, based on existing knowledge about the construct it is measuring” [33]. It consists of both convergent validity and known groups validity. Convergent validity assesses the correlation (or lack thereof) between constructs measured by two or more instruments, while known groups validity is an instrument’s ability to measure expected differences between subgroups of patients [33, 34]. All patients who completed the questionnaires at baseline and at one year were included in this analysis.

##### Convergent validity

For convergent validity, analysis at each time point is presented. (Analysis of the pooled HRQoL assessments is provided in additional file 1). The correlations between EPIC-26 domain scores and the dimensions of each of the utility based measures as well as the utility scores were examined using Spearman’s correlation coefficient. Statistical significance was considered at the 5% significance level. Correlations < 0.40 were considered weak, those between 0.40 and 0.70 were moderate, and those ≥ 0.70 were strong [35]. Strong correlations indicate that the measures or dimensions are measuring similar constructs. We hypothesised that there would be convergent validity in similar domains, such as EPIC-26 vitality/hormonal and sexual domains with vitality dimension on the SF-6D.

Modified Bland–Altman plots were also used to study further the limits of agreement between utility scores and the EPIC-26 domain scores. Because the instruments use different rating scales, leading to marked differences in the magnitude of the scores (i.e. 0–1 scale for SF-6D and AQoL-6D while EPIC-26 domain scores are on the 0–100 scale), standardised Z scores were calculated for the modified Bland–Altman plots. Similar to other studies, utilities and summary scores were power transformed to follow a normal distribution before calculating Z scores [36]. Good agreement was defined as only 5% of points being outside of the limits of agreement (LOA) [37].

To assess the agreement between individual measures (AQoL-6D, SF-6D and EPIC-26 individual domains), for each respondent, the intra-class correlation coefficient (ICC) was estimated using the z scores. The ICC was estimated using a two-way mixed-effect model with absolute agreement, where the individual effect was random, and the effect of the HRQoL measure was fixed. The strength of agreement was based on the following thresholds: ICC = 0–0.2 (poor),), ICC > 0.2–0.4 (fair),, ICC =  > 0.4–0.6 (moderate), ICC =  > 0.6–0.8 (strong) and ICC > 0.8 (almost perfect) [38].

##### Known groups validity

Known groups validity and was assessed using the Kruskal Wallis test. We hypothesised that there would be differences in HRQoL based on age at diagnosis [39], tumour/cancer stage [40, 41], and treatment group [40, 41]. Quality of life would be lower in the older age groups, in men with more advanced cancer and those receiving more invasive treatments such as radical prostatectomy compared to active surveillance or EBRT.

For all the analysis, a *p*-value of 0.05 was considered significant.

## Results

There was a total of 2587 patients, 1915 had complete data on all domains of the EPIC-26 and were included in this analysis. For both time points, 735 completed the EPIC-26, 206 completed both the EPIC-26 and AQoL-6D and 88 completed both EPIC-26 and the SF-12, none of the patients completed only AQoL-6D or SF-12. At baseline, 1454 completed EPIC-26 with 444 and 270 completing it in combination with the AQoL-6D and SF-12, respectively. At one year 1196 completed EPIC-26, with 598 and 437 completing in combination with AQoL-6D and SF-12 respectively. Table 1 presents the demographic and clinical characteristics of all the respondents who completed the questionnaires at baseline and at one year.

**Table 1:** Patient demographic characteristics

	**Completed assessments at**	
	**Baseline (*n* = 1454)**	**One year (*n* = 1196)**
**Age at diagnosis**		
**Mean (sd)**	65.3 (6.9)	64.9 (7.0)
**Median (IQR)**	66 (61, 70)	66 (61, 70)
	**N (%)**	**N (%)**
**Age categories**		
40–54	100 (6.9)	95 (7.9)
55–64	527 (36.2)	430 (36.0)
65–74	725 (49.9)	588 (49.2)
> = 75	102 (7.0)	83 (6.9)
Missing	0	0
**Tumour/cancer stage**		
T1	209 (14.4)	173 (14.5)
T2	868 (59.7)	682 (57.0)
T3/4	96 (6.6)	83 (6.9)
Missing	281 (19.3)	258 (21.6)
**Treatment**		
Active Surveillance	133 (9.2)	101 (8.4)
Radical prostatectomy	1120 (77.0)	891 (74.5)
Radiation therapy	141 (9.7)	148 (12.4)
Chemotherapy	1 (0.1)	4 (0.3)
Missing	59 (4.1)	52 (4.4)

*T1*  Tumour/Cancer stage 1, *T2*  Tumour/Cancer stage 2, *T3/4*  Tumour/Cancer stage 3 & 4

The mean age of the participants was 65 years, and the majority (38% at baseline and 31% at the one-year time point) were in the 65–74 age group. Most of the men (45% at baseline and 36% at one year) were diagnosed with stage T2 cancer and underwent radical prostatectomy (61% in baseline and 44% in one-year group).

Table 2 presents the descriptive statistics for health status. The mean (sd) utility scores at baseline were 0.86 (0.13) and 0.81 (0.12) for AQoL-6D and SF-6D, respectively and 0.86 (0.14) and 0.82 (0.12) at one-year. Summary scores for the EPIC domains are shown in Table 2.

**Table 2:** Descriptive statistics for health status

	**n**	**Mean**	**SD**	**Median**	**IQR**
**AQoL-6D**					
Baseline	444	0.86	0.13	0.89	0.82, 0.94
1 year	598	0.86	0.14	0.90	0.81, 0.96
**SF-6D**					
Baseline	270	0.81	0.12	0.86	0.72, 0.92
1 year	437	0.82	0.12	0.86	0.74, 0.92
**EPIC-26**					
**Baseline**					
Urinary Incontinence	1454	91.5	15.7	100	85.5, 100
Urinary Irritation/Obstruction	1454	86.1	15.1	90.6	81.3, 100
Bowel	1454	93.7	10.9	100	91.6, 100
Sexual	1454	61.7	30.8	69.5	36.2, 87.5
Vitality/Hormonal	1454	94.0	9.1	100	90, 100
**One year**					
Urinary Incontinence	1196	79.0	22.7	85.5	64.8, 100
Urinary Irritation/Obstruction	1196	91.2	11.6	93.8	87.5, 100
Bowel	1196	92.7	12.4	100	91.7, 100
Sexual	1196	33.2	28.0	22.2	12.5, 54.2
Vitality/Hormonal	1196	90.5	13.4	95	85, 100

### Responsiveness

AQoL-6D utility scores deteriorated, while those for the SF-6D improved between baseline and one year. However, the mean change in utility scores for both measures was not statistically significant (Table 3). Both effect size and SRM values for both measures were below 0.2, indicating low sensitivity to change. All EPIC domains deteriorated at one year, except for urinary irritation/obstruction, which improved. The mean changes in all EPIC domains were statistically significant, except for the bowel domain. The changes in urinary incontinence, urinary obstruction and sexual domains were clinically significant as they were above the MCID at 12.5, 5.1 and 28.5 points, respectively. All domains demonstrated low sensitivity to change except for urinary incontinence, which was moderately sensitive (ES = 0.65 and SRM = 0.46) and the sexual domain with high sensitivity to change (ES = 0.96 and SRM = 1.04).

**Table 3:** Responsiveness between baseline and 1 year

**Items**	**N**	**Baseline (mean)**	**One year (mean)**	**Mean change**	**SD at baseline**	**SD of change**	**ES**	***p*-value**	**SRM**
AQoL-6D utility score	206	0.87	0.86	0.01	0.12	0.18	0.10	0.32	-0.07
SF-6D utility score	88	0.8	0.82	-0.02	0.13	0.18	-0.19	0.21	0.13
Urinary Incontinence	735	91.4	79.3	**12.13**	16.6	27.93	0.61**	**0.00**	-0.43*
Urinary Irritation/Obstruction	735	86.5	92.2	**-5.71**	15.4	18.46	-0.44*	**0.00**	0.31*
Bowel	735	94.2	93.7	0.56	10.5	15.77	0.05	0.33	-0.04
Sexual	735	64.6	36.9	**27.68**	30	41.81	0.94***	**0.00**	-0.66**
Vitality/Hormonal	735	93.9	91.5	0.01	9.23	15.00	0.22*	0.32	-0.16

*SD* Standard deviation; ES effect size = (mean change/SD at baseline) *Small change, small responsiveness **Moderate change, moderately responsive ***Large change, largely responsive; SRM standardized response mean = (mean change/SD of change). If SRM = 0.2 to 0.49—small, 0.50 to 0.79 equals moderate and 0.80 and above equals large; Minimum clinically important difference (MCID) range: Bowel and Vitality/hormonal domains = 4–6 point change; Sexual domain = 10–12; Urinary incontinence = 6–9; Urinary irritation/obstruction domain = 5–7

Bold mean change = MCID achieved, clinically significant; Bold *p*-value = statistically significant

### Convergent validity of EPIC-26 with AQoL-6D and SF-6D

Table 4 shows the correlations between EPIC-26 domains and the dimensions of the AQoL-6D and SF-6D. Moderate evidence of convergent validity was observed between EPIC-26 sexual and vitality/hormonal domain with relationships and coping on AQoL-6D at both time points. The vitality/hormonal domain was also moderately correlated with independent living (*r* = 0.40) and mental health (*r *= 0.43) at one year. With SF-6D, the EPIC-26 vitality/hormonal domain showed moderate convergent validity with the role and social function at both baseline and one year. Weak correlations (*r* < 0.40) were observed between all other EPIC domains and the dimensions of the generic measures.

**Table 4:** Correlations between EPIC domains and utility and domain scores (Spearman)

**Generic QoL domains**	**EPIC-26 domains**									
	**Baseline**					**1 Year**				
	Urinary Incontinence	Urinary Irritation/Obstruction	Bowel	Sexual	Vitality/ Hormonal	Urinary Incontinence	Urinary Irritation/Obstruction	Bowel	Sexual	Vitality/ Hormonal
**AQoL domains**										
AQoL utility score	0.22	0.31	0.31	0.32	**0.45**^a^	0.28	0.34	0.37	0.34	**0.53**^a^
Independent Living	0.23	0.34	0.30	0.33	0.39	0.20	0.32	0.29	0.33	**0.40**^a^
Relationships	0.18	0.22	0.21	**0.41**^a^	0.36	0.24	0.20	0.22	**0.41**^a^	0.33
Mental Health	0.13	0.21	0.18	0.14	0.31	0.24	0.28	0.28	0.17	**0.43**^**a**^
Coping	0.18	0.23	0.24	0.29	**0.46**^a^	0.21	0.26	0.34	0.27	**0.54**^a^
Pain	0.23	0.28	0.31	0.13	0.26	0.17	0.28	0.28	0.21	0.32
Senses	0.16	0.15	0.18	0.17	0.17	0.14	0.16	0.19	0.16	0.19
**SF-6D domains**										
SF-6D utility score	0.19	0.27	0.33	0.28	**0.52**^a^	0.31	**0.40**^a^	0.28	0.25	**0.57**^a^
Physical function	0.19	-	0.14	0.17	-	-	0.09	-	-	-
Role	0.22	0.18	0.25	0.31	**0.41**^a^	0.25	0.28	0.25	0.22	**0.50**^a^
Social	0.15	0.18	0.26	0.20	**0.47**^a^	0.26	0.34	0.24	0.16	**0.50**^a^
Pain	0.23	0.21	0.23	0.18	0.24	-	0.18	0.18	0.11	0.31
Mental health	-	0.17	0.20	0.13	0.35	0.21	0.28	0.17	0.14	0.37
Vitality	0.15	0.15	0.22	0.17	0.27	0.11	0.19	0.14	0.19	0.30

Only significant (at 5% level of significance) correlations showed. Correlations < 0.40 were considered weak, those between 0.40 and < 0.70 were moderate(^a^), and ≥ 0.70 were strong [35]

**Fig. 1:**
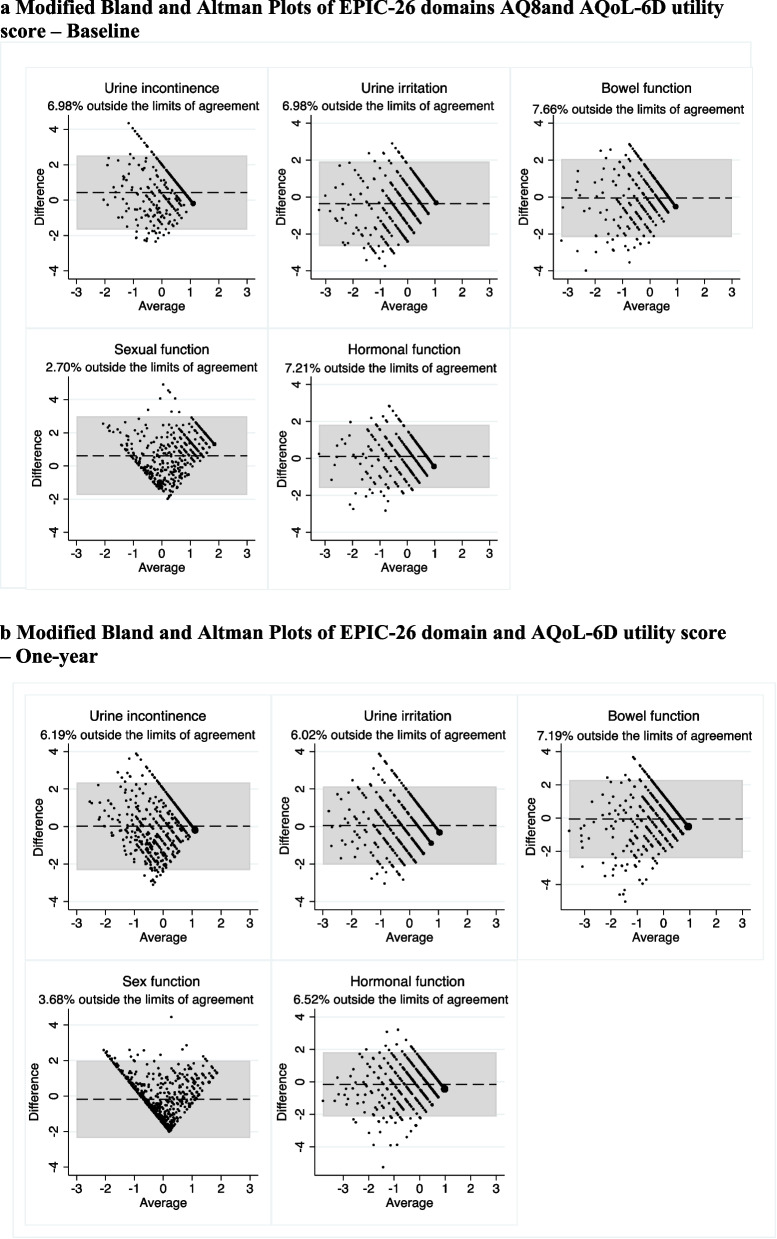
**a** Modified Bland and Altman Plots of EPIC-26 domains and AQoL-6D utility score – Baseline. **b** Modified Bland and Altman Plots of EPIC-26 domain and AQoL-6D utility score – One-year

**Fig. 2:**
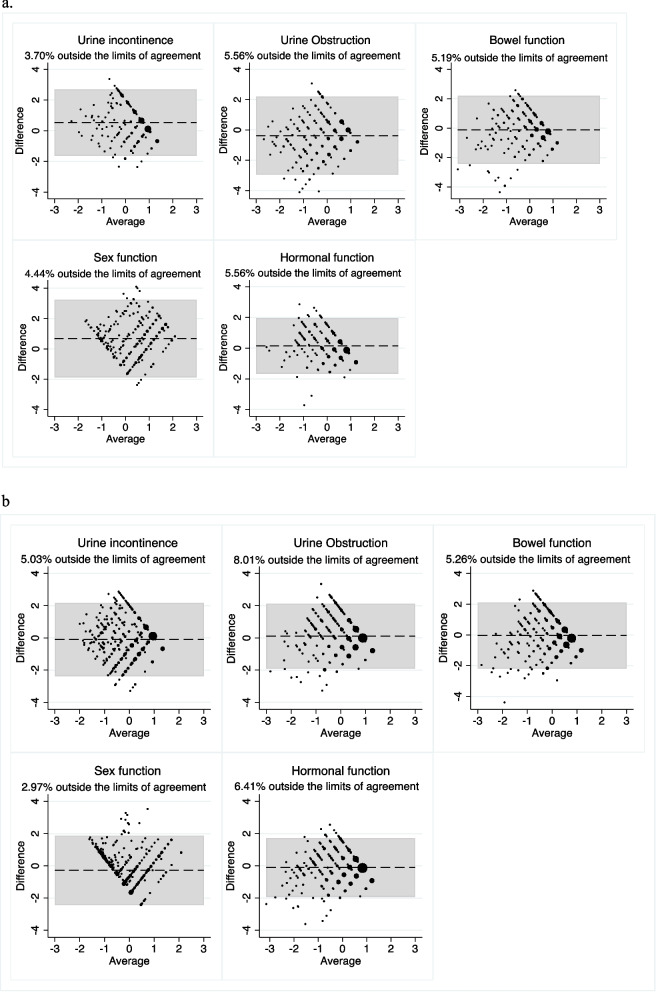
**a** Modified Bland and Altman Plots of EPIC-26 domain and SF-6D utility score – Baseline. **b** Modified Bland and Altman Plots of EPIC-26 domain and SF-6D utility score – One year

The modified Bland–Altman plots show the limits of agreement between EPIC-26 domains and AQoL-6D (Fig. 1a and b) and SF-6D (Fig. 2a and b) utility scores. A weak agreement was observed between the utility scores and most EPIC-26 domains. The sexual domain showed good agreement with both AQoL-6D and SF-6D utility scores at both time points. Good agreement was also observed between SF-6D utility score and urinary incontinence domain at baseline but not at one year.

Results of the intraclass correlation are presented in Table 5.

**Table 5:** Intra-class correlation

**EPIC and AQoL-6D**	**Baseline, *n* = 444**		**1 year, *n* = 598**	
	**Individual**	**Average**	**Individual**	**Average**
ICC	0.27	0.69	0.31	0.73
95% CI	0.22 – 0.32	0.62 – 0.74	0.27 – 0.34	0.69 – 0.76
**EPIC and SF-6D**	**Baseline, *n* = 270**		**1 year, *n* = 437**	
	**Individual**	**Average**	**Individual**	**Average**
ICC	0.25	0.67	0.28	0.70
95% CI	0.19 – 0.32	0.58 – 0.74	0.24 – 0.32	0.66 – 0.74

The strength of agreement was based on the following thresholds: ICC = 0–0.2 (poor), ICC = 0.2–0.4 (fair), ICC = 0.4–0.6 (moderate), ICC = 0.6–0.8 (strong) and ICC > 0.8 (almost perfect) [38, 42]

The absolute agreement between individual AQoL-6D utility scores and the domain scores of the EPIC-26 was low at baseline (0.27) and one year (0.31). Similarly, the agreement between the EPIC-26 and the SF-6D utility score was low at baseline (0.25) and one year (0.28). But the agreement between the mean scores was strong at 0.69 and 0.73 with AQoL-6D and 0.67 and 0.70 with SF-6D at baseline and one year, respectively.

### Known groups validity

Table 6 shows the results of the known groups validity. Both AQoL-6D and SF-6D did not discriminate between age groups and tumour stages at both time points. Only AQoL-6D discriminated between treatment groups at one year. All EPIC-26 domains discriminated between age groups at both time points except the vitality/hormonal domain. Urinary incontinence and bowel domains did not discriminate between tumour stage, while all domains discriminated between treatment groups.

**Table 6:** Known groups validity between age groups, tumour/cancer stage and treatment [n, mean (sd)]

	**Baseline**					**1 year**				
**HRQoL**	**Age group**									
	**40–54**	**55–64**	**65–74**	** ≥ 75**	***p*-value**	**40–54**	**55–64**	**65–74**	** ≥ 75**	***p*-value**
AQoL-6D utility score	*N* = 17 0.83 (0.11)	*N* = 138 0.85 (0.14)	*N* = 235 0.87 (0.13)	*N* = 54 0.87 (0.10)	0.084	*N* = 36 0.88 (0.15)	*N* = 186 0.85 (0.16)	*N* = 319 0.87 (0.13)	*N* = 57 0.82 (0.16)	0.063
SF-6D utility score	*N* = 25 0.80 (0.12)	*N* = 96 0.81 (0.14)	*N* = 127 0.82 (0.11)	*N* = 22 0.83 (0.12)	0.775	*N* = 41 0.84 (0.10)	*N* = 171 0.81 (0.12)	*N* = 202 (0.83 (0.11)	*N* = 23 0.84 (0.12)	0.268
**EPIC domain**	***N* = *100***	***N* = *527***	***N* = *725***	***N* = *102***		***N* = *95***	***N* = *430***	***N* = *588***	***N* = *83***	
Urinary Incontinence	94.8 (11.0)	93.2 (14.3)	90 (17.1)	89.9 (14.8)	** < *0.001***	81.8 (21.2)	81.6 (21.8)	76.4 (23.4)	81.9 (23.0)	** < *0.001***
Urinary Irritation/Obstruction	89.6 (16)	87.2 (14.3)	85.3 (15.2)	83 (15.0)	** < *0.001***	92.4 (10.4)	92.0 (12.0)	90.6 (11.5)	90.1 (11.1)	***0.019***
Bowel	94.8 (12.1)	94.4 (10.2)	93.3 (11.1)	92.0 (12.7)	***0.009***	94.3 (12.5)	93.1 (12.5)	92.6 (12.1)	89.6 (13.5)	***0.002***
Sexual	80.5 (21.5)	70.1 (28.1)	55.8 (30.9)	41.8 (29.7)	** < *0.001***	54.7 (28.2)	39.6 (29.3)	26.6 (24.8)	22.4 (21.6)	** < *0.001***
Vitality/Hormonal	93.3 (9.6)	94.1 (9.2)	94.1 (8.9)	93.8 (9.4)	0.899	91.9 (11.9)	89.8 (14.0)	90.7 (13.5)	89.9 (10.9)	0.287
**HRQoL**	**Tumour/cancer stage**									
	**T1**	**T2**	**T3/4**		***p*-value**	**T1**	**T2**	**T3/4**		***p*-value**
AQoL-6D utility score	*N* = 61 0.86 (0.12)	*N* = 335 0.86 (0.12)	*N* = 27 0.84 (0.17)		0.749	*N* = 96 0.87 (0.14)	*N* = 409 0.86 (0.14)	*N* = 45 0.82 (0.18)		0.220
SF-6D utility score	*N* = 22 0.81 (0.14)	*N* = 123 0.81 (0.13)	*N* = 19 0.82 (0.11)		0.982	*N* = 54 0.83 (0.11)	*N* = 208 0.82 (0.12)	*N* = 30 0.82 (0.10)		0.810
**EPIC domain**	***N* = *209***	***N* = *868***	***N* = *96***			***N* = *173***	***N* = *682***	***N* = *83***		
Urinary Incontinence	92.2 (15.0)	91.3 (16.1)	91.4 (15.2)		0.919	81.6 (20.2)	79.5 (22.6)	77.8 (27.0)		0.725
Urinary Irritation/Obstruction	88.1 (14.1)	85.2 (15.7)	84.4 (15.6)		***0.023***	93.1 (10.2)	90.0 (11.6)	89.2 (13.1)		***0.016***
Bowel	94 (11.2)	93.7 (11.1)	91.8 (14.0)		0.117	94.1 (11.1)	92.2 (13.5)	91.8 (11.0)		0.103
Sexual	62.9 (31.4)	61.6 (30.5)	56.6 (32.2)		0.219	34.7 (28.7)	34.1 (28.0)	24.4 (24.5)		***0.004***
Vitality/Hormonal	94.6 (9.1)	93.8 (9.2)	93.1 (11.1)		0.299	92 (11.2)	90.5 (13.1)	84 (19.7)		***0.016***
**HRQoL**	**Treatment group**									
	**A**	**R**	**RadTx**	**Chemo**	***p*-value**	**A**	**R**	**RadTx**	**ChemoTx**	***p*-value**
AQoL-6D utility score	*N* = 78 0.86 (0.12)	*N* = 257 0.87 (0.12)	*N* = 63 0.85 (0.14)	*N* = 1 1.0	0.366	*N* = 75 0.84 (0.14)	*N* = 383 0.87 (0.14)	*N* = 89 0.83 (0.16)	*N* = 3 0.86 (0.08)	0.099
SF-6D utility score	*N* = 33 0.81 (0.13)	*N* = 158 0.81 (0.12)	*N* = 72 0.82 (0.12)	*N* = 0	0.913	*N* = 24 0.77 (0.14)	*N* = 354 0.83 (0.12)	*N* = 54 0.82 (0.12)	*N* = 1 0.79	0.174
**EPIC domain**	***N* = *133***	***N* = *1120***	***N* = *141***	***N* = *1***		***N* = *101***	***N* = *891***	***N* = *148***	***N* = *4***	
Urinary Incontinence	91.2 (16.9)	92.0 (15.5)	89.4 (15.4)	100	***0.028***	82.1 (21.4)	76.8 (23.2)	87.7 (18.3)	96.4 (7.3)	** < *0.001***
Urinary Irritation/Obstruction	85.6 (14.7)	86.8 (14.7)	82.4 (16.2)	93.8	***0.002***	87.4 (15.4)	93.0 (9.7)	83.4 (15.1)	90.6 (6.3)	** < *0.001***
Bowel	93.0 (9.7)	94.3 (10.5)	89.9 (13.6)	91.7	** < *0.001***	92.0 (13.2)	93.6 (11.9)	87.5 (14.6)	87.5 (10.2)	** < *0.001***
Sexual	61.6 (30.1)	63.6 (30.2)	48.3 (32.2)	87.5	** < *0.001***	42.4 (29.9)	32.1 (27.4)	33.7 (28.6)	24.0 (17.4)	***0.012***
Vitality/Hormonal	93.7 (8.2)	94.5 (8.6)	92.1 (11.0)	95	***0.032***	90.5 (12.8)	91.7 (12.1)	84.2 (17.9)	75 (9.1)	** < *0.001***

*T1*  Tumour stage 1, *T2*  Tumour stage 2, *T3/4*  Tumour stage 3 and 4; Bold *p*-value = statistically significant

Treatment groups: A = Active surveillance, R = Radical prostatectomy, RadTx = Radiation ChemoTx = chemotherapy

## Discussion

The past decade has seen an increase in the development of condition-specific patient-reported outcome measures for cancer [10, 24, 43]. However, their use in decision-making within the discipline of health economics remains limited. This study empirically compared a prostate cancer-specific patient-reported outcome measure (EPIC-26) against two generic utility measures (AQoL-6D and SF-6D) using a sample drawn from a longitudinal cohort of men diagnosed with prostate cancer between February 2010 and March 2020. The study analysed HRQoL outcomes at baseline and one-year post-treatment. Across Australia, radical prostatectomy is reported in 50% of newly diagnosed prostate cancer cases [44] and our dataset did not deviate from this expected proportion with majority of the men undergoing prostatectomy (77%) and 10% each on active surveillance and radiation therapy.

EPIC-26 domain scores reduced over time, similar to other longitudinal studies applying the EPIC-26 in a similar population [45, 46]. When assessing responsiveness the generic measures were not expected to be sensitive to change [32], but high sensitivity and responsiveness were expected for the EPIC-26 domains [24, 46]. However, the EPIC-26 effect size and SRM were small except for the sexual domain.

Although it is recommended that EPIC-26 is administered in combination with the generic SF-12 [47], no studies have investigated the convergent validity of EPIC-26 domains with the domains of the SF-12 or its preference-based SF-6D. This study showed positive correlations between AQoL-6D and SF-6D utility scores and all EPIC-26 domains. Utility scores for both generic measures were moderately correlated with the hormonal domain but weakly correlated with all the other domains. Similarly, for the individual dimensions of AQoL-6D and SF-6D, the vitality/hormonal domain was moderately correlated with independent living, mental health and coping on AQoL-6D, and with the role and social function on SF-6D. Studies have shown that, treatment for prostate cancer is associated with depression and poorer HRQoL due to mental factors [48, 49]. It is therefore not surprising that the vitality/hormonal domain, although not strongly correlated with the mental health domain of both AQoL and SF-6D, was correlated with nearly all domains of both measures. The breadth of dimensions this domain is correlated with may imply the far-reaching effects of hormonal impairment on overall quality of life.

As with other studies investigating convergent validity between generic and condition-specific instruments [19, 36, 43], we hypothesised that EPIC-26 domains would be highly correlated with AQoL-6D and SF-6D dimensions that measure similar constructs, such as the sexual domain with vitality on the SF-6D, but this correlation was weak. However, the sexual domain was moderately correlated with the relationship dimension of AQoL-6D and the Bland Altman plots showed strong agreement between both utility scores and the sexual domain at both time points.

Both generic measures did not discriminate between age groups and tumour stage, while only AQoL-6D discriminated treatment groups and is recommended for assessing HRQoL outcomes following treatment. All EPIC domains discriminated between age groups (except the vitality/hormonal domain) and treatment group, but only one domain discriminated between tumour stage at baseline and three domains at one year. Being disease-specific, we hypothesised that EPIC-26 domains would discriminate between both tumour stage and treatment groups, but this was only observed for the latter. It can be argued, therefore, that disease-specific EPIC-26 domains are more suitable for discriminating in outcomes following treatment.

It is also important to note that similar to the generic measures, only the vitality/hormonal domain did not discriminate between age groups. This observation, in addition to the moderate correlations between this domain and the utility scores noted above, may suggest a possible relationship between vitality/hormonal and overall quality of life.

In addition to previous studies, this study supports the utility of the disease-specific EPIC-26 in cost-effectiveness analysis, a type of economic evaluations. All three instruments comparably discriminate between general characteristics, but the disease-specific measure is better at discriminating between disease-specific characteristics and post-treatment outcomes. For purposes of economic evaluation, the choice of instrument(s) depends on the questions being asked and the outcomes of interest. If urinary incontinence (leakage incidents, use of pads) is the outcome of interest, then the EPIC-26 has to be used. Even if SF-6D and AQoL-6D scores correlated highly with the urinary incontinence scores, these measures do not elicit enough information on the disease-specific impairments. On the other hand, if utility scores are needed for generating QALYs in a health economic model, knowing how many pads the patients used may be less important.

The developers of the EPIC-26 recommend that it is used in combination with SF-12 to provide a holistic assessment of HRQoL. Both AQoL-6D and SF-6D were comparable in discriminating between disease-specific characteristics, but only AQoL-6D could discriminate between outcomes following treatment. Results from this study posit the AQoL-6D as a potential (maybe even better) compliment to EPIC-26. This proposition requires further study with a larger sample size.

The main strength of this study is that it provides the first head-to-head empirical assessments of the construct validity and responsiveness of two generic utility measures, AQoL-6D and SF-6D, relative to the EPIC-26. However, the main weakness was that while data on the EPIC-26 vs AQoL-6D and EPIC-26 vs SF-6D head-to-head comparisons were collected, none were collected for the AQOL-6D vs SF-6D correlation. Consequently, head-to-head comparisons between the AQoL-6D and SF-6D were not possible.

## Conclusion

This study provides insights into whether generic preference-based measures of HRQoL can be used interchangeably or as a complement to the disease-specific EPIC-26. All three instruments are comparable in discriminating between general characteristics, but the disease-specific measure is better at discriminating in disease-specific characteristics and post-treatment outcomes. Using the disease-specific EPIC-26 and a generic measure provides a holistic assessment of quality of life in this population. In contrast, the generic measure is suitable for generating QALYs for use in CUA.

## Supplementary Information

**Additional file 1.**

## Data Availability

Data can be made available on request from the Corresponding author.

## Abbreviations

AQoL-6D: Assesment of Quality of Life 6 Dimensions
CEA: Cost Effectiveness Analysis
CUA: Cost Utility Analysis
EPIC-26: Expanded Prostate Cancer Index Composite Instrument
ES: Effect Size
HRQoL: Health Related Quality of Life
ICC: Intra-class correlation coefficient
LOA: Limits of Agreement
MAUIs: Multi-Attribute Utility Instruments
MCID: Minimum clinically important difference
MCS: Mental Component Summary
MSAC: Medical Services Advisory Committee
NICE: National Institute for Health and Care Excellence
PBAC: Pharmaceutical Benefits Advisory Committee
PCS: Physical Component Summary
QALYs: Quality Adjusted Life Years
QLU-C10D: Quality of Life Utility Measure-Core 10 dimensions
SA-PCCOC: South Australian Prostate Cancer Clinical Outcomes Collaborative
SF-12: Short Form Health Survey
SF-6D: Short Form 6 Dimensions
SRM: Standardized Response Mean
