# Supplementary material for: Responsiveness and construct validity of EPIC-26, AQoL-6D and SF-6D following treatment in prostate cancer
Source: BMC Cancer. 2023 Apr 1;23:297. doi: 10.1186/s12885-023-10732-6 (PMC10067207; doi:10.1186/s12885-023-10732-6)
Supplement: Supplementary file 1 — Additional file 1. [file 12885_2023_10732_MOESM1_ESM.docx]

**Additional file**

**Article title: Construct validity and responsiveness of EPIC-26, AQoL-6D and SF-6D following treatment for prostate cancer**

**Authors**: Norma B. Bulamu^1^*, Christine Mpundu-Kaambwa^2^, Michael O'Callaghan^1,3,4^, Billingsley Kaambwa^1^

^1^ Flinders Health and Medical Research Institute, College of Medicine and Public Health, Flinders University, Adelaide, Australia

^2^ Health and Social Care Economics Group, College of Nursing and Health Sciences, Flinders University, Adelaide, Australia

^3^ SA-PCCOC: South Australian Prostate Cancer Clinical Outcomes Collaborative, Flinders Medical Centre, Urology Unit, Adelaide, Australia

^4^ University of Adelaide, Discipline of Medicine^,^ Adelaide, Australia

***Corresponding author:**

Dr Norma B. Bulamu

Flinders Health and Medical Research Institute

College of Medicine and Public Health

Flinders University

Adelaide, South Australia

Telephone: +61 8 74219956

Email: [norma.bulamu@flinders.edu.au](mailto:norma.bulamu@flinders.edu.au)

**Supplementary data: Results from pooled analysis of baseline and 1-year responses**

### Figure S1: Correlation between the EPIC-26 domain and AQoL-6D utility scores

### Figure S2: Correlation between the EPIC-26 domain and SF-6D utility scores

### Figure S3: Modified Bland and Altman Plot of EPIC-26 domain and AQoL-6D utility score

### Figure S4: Modified Bland and Altman Plot of EPIC-26 domain and SF-6D utility score

### Table S1: Correlations (Spearman) – both timepoints

| **Generic QoL domains** | **EPIC-26 domains** | | | | |
| --- | --- | --- | --- | --- | --- |
|  | Urinary Incontinence | Urinary Irritation/Obstructive | Bowel | Sexual | Vitality/  Hormonal |
| **AQoL domains** | | | | | |
| AQoL utility score | 0.25 | 0.32 | 0.35 | 0.31 | **0.50***** |
| Independent Living | 0.22 | 0.32 | 0.29 | 0.32 | 0.39 |
| Relationships | 0.26 | 0.17 | 0.21 | **0.44*** | 0.36 |
| Mental Health | 0.17 | 0.27 | 0.24 | 0.11 | 0.37 |
| Coping | 0.21 | 0.24 | 0.30 | 0.28 | **0.51**** |
| Pain | 0.19 | 0.27 | 0.29 | 0.17 | 0.29 |
| Senses | 0.15 | 0.15 | 0.18 | 0.16 | 0.18 |
| **SF-6D domains** | | | | | |
| SF-6D utility score | 0.25 | 0.34 | 0.30 | 0.23 | **0.54**** |
| Physical function | 0.08 | 0.11 | 0.08 | 0.11 | 0.08 |
| Role | 0.21 | 0.24 | 0.26 | 0.22 | **0.46*** |
| Social | 0.20 | 0.27 | 0.25 | 0.14 | **0.48*** |
| Pain | 0.12 | 0.17 | 0.20 | 0.14 | 0.29 |
| Mental health | 0.13 | 0.23 | 0.18 | 0.12 | 0.36 |
| Vitality | 0.13 | 0.15 | 0.17 | 0.19 | 0.29 |

*All correlations were significant at 5% level of significance. *Strong correlation. Spearman correlation 0.30=weak, 0.40 - 0.50 = moderate, and above 0.50 = strong*

### Table S2: Intra-class correlation – both timepoints

|  | **Individual** | **Average** |
| --- | --- | --- |
| **EPIC and AQoL-6D: n=1042** | | |
| ICC | 0.29 | 0.71 |
| 95% CI | 0.26 - 0.32 | 0.68 - 0.74 |
| **EPIC and SF-6D: n=712** |  |  |
| ICC | 0.26 | 0.68 |
| 95% CI | 0.23 - 0.30 | 0.65 - 0.72 |

### Table S3: Discriminant validity between age groups tumour/cancer stage and treatment [n, mean (sd)]

|  | **Age group** | | | | | | | | |
| --- | --- | --- | --- | --- | --- | --- | --- | --- | --- |
| **HRQoL or domain score** |  | **40-54** |  | **55-64** |  | **65-74** |  | **≥ 75** | **p-value** |
| AQoL-6D utility score | 53 | 0.86 (0.14) | 324 | 0.85 (0.15) | 554 | 0.87 (0.13) | 111 | 0.84 (0.13) | 0.11 |
| SF-6D utility score | 66 | 0.83 (0.11) | 270 | 0.82 (0.13) | 331 | 0.82 (0.11) | 45 | 0.83 (0.12) | 0.51 |
| Urinary Incontinence | 195 | 88.5 (17.9) | 960 | 87.9 (19.1) | 1315 | 83.9 (21.3) | 185 | 86.3 (19.3) | ***0.0001*** |
| Urinary Irritative/Obstructive | 195 | 91 (13.6) | 960 | 89.3 (13.8) | 1315 | 87.7 (13.9) | 185 | 86.2 (13.8) | ***0.0001*** |
| Bowel | 195 | 94.6 (12.3) | 960 | 93.8 (11.3) | 1315 | 93 (11.4) | 185 | 90.9 (13.1) | ***0.0001*** |
| Sexual | 195 | 67.9 (28.1) | 960 | 56.3 (32.4) | 1315 | 42.7 (31.8) | 185 | 33.1 (28.0) | ***0.0001*** |
| Vitality/Hormonal | 195 | 92.6 (10.8) | 960 | 92.2 (11.8) | 1315 | 92.6 (11.2) | 185 | 92.1 (10.3) | 0.73 |
|  | **Tumour/cancer stage** | | | | | | | | |
| **HRQoL or domain score** |  | **T1** |  | **T2** |  | **T3/4** |  |  | **p-value** |
| AQoL-6D utility score | 157 | 0.87 (0.14) | 744 | 0.86 (0.13) | 72 | 0.83 (0.17) |  |  | 0.21 |
| SF-6D utility score | 76 | 0.82 (0.12) | 335 | 0.82 (0.12) | 49 | 0.82 (0.11) |  |  | 0.86 |
| Urinary Incontinence | 382 | 86.4 (18.3) | 1554 | 86.1 (20.1) | 179 | 85.1 (22.5) |  |  | 0.77 |
| Urinary Irritation/Obstruction | 382 | 90.4 (12.8) | 1554 | 87.7 (14.3) | 179 | 86.6 (14.7) |  |  | ***0.001*** |
| Bowel | 382 | 94.0 (11.1) | 1554 | 93.1 (12.2) | 179 | 91.8 (12.7) |  |  | ***0.013*** |
| Sexual | 382 | 50.1 (33.3) | 1554 | 49.5 (32.4) | 179 | 41.7 (33.0) |  |  | ***0.005*** |
| Vitality/Hormonal | 382 | 93.4 (10.2) | 1554 | 92.4 (11.2) | 179 | 88.9 (16.3) |  |  | ***0.019*** |
|  |  |  |  | **Treatment group** |  |  |  |  |  |
| **HRQoL or domain score** | **Active Surveillance** | | **Radical prostatectomy** | | **Non-surgical treatment (Radiation and chemotherapy)** | | |  | **p-value** |
| AQoL-6D utility score | 95 | 0.86 (0.12) | 642 | 0.87 (0.12) | 221 | 0.83 (0.16) |  |  | ***0.009*** |
| SF-6D utility score | 25 | 0.83 (0.13) | 495 | 0.82 (0.12) | 181 | 0.82 (0.12) |  |  | 0.76 |
| Urinary Incontinence | 124 | 92.8 (13.5) | 2004 | 85.8 (20.4) | 425 | 84.1 (20.8) |  |  | ***0.0001*** |
| Urinary Irritation/Obstruction | 124 | 86.8 (14.0) | 2004 | 89.4 (13.3) | 425 | 84.6 (15.1) |  |  | ***0.0001*** |
| Bowel | 124 | 92.9 (11.6) | 2004 | 94.6 (10.2) | 425 | 87.4 (15.6) |  |  | ***0.0001*** |
| Sexual | 124 | 60.4 (28.9) | 2004 | 50.8 (32.8) | 425 | 37.0 (30.9) |  |  | ***0.0001*** |
| Vitality/Hormonal | 124 | 93.1 (9.0) | 2004 | 93.7 (9.7) | 425 | 87.2 (16.0) |  |  | ***0.0001*** |

*T1=Tumour stage 1, T2=Tumour stage 2, T3/4=Tumour stage 3 and 4;* ***Bold p-value = statistically significant***
